# Do people with bipolar disorder have a lack of empathy?

**DOI:** 10.1192/j.eurpsy.2022.1060

**Published:** 2022-09-01

**Authors:** E. Mhiri, J. Ben Thabet, R. Feki, I. Gassara, N. Smaoui, L. Zouari, S. Omri, M. Maalej, N. Charfi, M. Maalej

**Affiliations:** 1 Psychiatry C Department, Hedi Chaker, sfax, Tunisia; 2 psychiatry department, Hedi Chaker, sfax, Tunisia

**Keywords:** empathy, bipolar, euthymic, matched, controls

## Abstract

**Introduction:**

Impairments of empathy have been observed in patients with various psychiatric Disorders. Yet, little research on empathy concerning mood disorders exists.

**Objectives:**

To compare empathy levels in euthymic bipolar patients (BP) and healthy controls (HC).

**Methods:**

A cross-sectional and comparative study of 78 patients followed for bipolar disorder, during euthymia, at the psychiatric outpatient clinic at CHU Hédi Chaker in Sfax, and 78 age-gender matched HC. We used a socio-demographic and clinical data sheet and the Questionnaire of Cognitive And Affective Empathy (QCAE) to assess empathy with its two dimensions : “Affective empathy” and “Cognitive empathy”.

**Results:**

The average age was 36.27 years, the sex ratio was 5.5. Bipolar I disorder was diagnosed in 88.5% of patients. The mean age of onset was 27.73 years, and the mean duration of illness was 8.4 years. Total scores of empathy as well as scores of cognitive and affective empathy were higher in HC than in BP. *Total QCAE BP vs HC : 72.49 vs 80.53 *Cognitive empathy BP vs HC : 43.21 vs 94.24 *Affective empathy BP vs HC : 29.36 vs 30.44 A significant difference in QCAE score and cognitive empathy score between BP and HC was found (p<10-3).

**Conclusions:**

In our study, euthymic BP have been less empathetic than HC. Research on the subject are small and few. Thus, more studies are needed to confirm our results on the effect of mood disorders on empathy.

**Disclosure:**

No significant relationships.

